# Exploring personality correlates of falsification of COVID-19 lateral flow tests through vignettes

**DOI:** 10.1177/13591053241298034

**Published:** 2024-11-25

**Authors:** Devashish Ray, Raenhha Dhami, Aritra Mukherjee, Jan Lecouturier, Laura J McGowan, Ivo Vlaev, Michael P Kelly, Falko F Sniehotta

**Affiliations:** 1 Newcastle University, UK; 2 Heidelberg University, Germany; 3 University of Warwick, UK; 4 University of Cambridge, UK

**Keywords:** COVID-19, individual differences, personality traits, prosocial behaviour, vignettes

## Abstract

Regular testing using rapid antigen lateral flow tests (LFTs) was an important prevention strategy during the COVID-19 pandemic. However, falsification of home LFTs was a concern. Using a large quota-representative sample of adults (*n* = 1295) in England, we conducted a vignette survey consisting of four hypothetical scenarios of LFT falsification behaviours (FBs) to examine whether prosocial personality traits were associated with attitudes towards and intentions for falsifying home LFTs during the pandemic. Results show that higher levels of affective empathy for people vulnerable to COVID-19 and Honesty-Humility from the HEXACO model of Personality are associated with non-acceptability of LFT FBs and intentions to not engage with them. However, affective empathy explained more of the variation compared to the facet-level measures of Honesty-Humility for both attitude and intention. Public health communications aimed at encouraging personal protective behaviours should consider the role of empathy and prosocial messages.

## Introduction

Large-scale home testing using lateral flow tests (LFTs) was one strategy used to contain the spread of the COVID-19 virus in England, and globally (WHO, 2022). Between April 2021 and March 2022, everyone in England was provided access to free LFTs. The government urged the public to use LFTs for twice-a-week testing, even when asymptomatic, and to report results of all tests (irrespective of the result) either online through a government website or through a 24-hour phone helpline (DHSC, 2021). Those who tested positive were required to self-isolate immediately. Proof of a recent (taken within 48 hours) negative result was introduced as a requirement to gain access to work and educational settings, to indoor and outdoor mass events and for travel. Regular self-testing was promoted as a civic and moral duty to family, friends and the community. However, complying with guidance on testing placed a significant social, economic and practical burden on the individual and sometimes raised social and ethical dilemmas (Street et al., 2022). As the use of LFTs became widespread practice, there were media reports of some falsification of LFT results, for example, to gain entry to mass events (O’Leary, 2021), to avoid travel restrictions (Kelleher, 2021) or to avoid school or work (Hudspith, 2022). The possibility that falsification of home LFTs could hamper efforts to control the pandemic was a potential public health problem (Deguma and Deguma, 2021).

Compliance with guidance for self-testing using LFTs and various COVID-19 transmission-reducing behaviours (TRBs; e.g. physical distancing, hand washing, wearing a face covering, self-isolation) can be considered as pro-social behaviours, in the sense that one sacrifices certain comforts and obligations for the overall good of others. Concurrently, such compliance protects both oneself and the most vulnerable (De Cremer and Van Lange, 2001). Behavioural and social science research has consistently demonstrated that individual differences in prosocial behaviours are determined by personality, situational factors and their interaction (Thielmann et al., 2020). Personality refers to a set of traits that are present in an individual from an early age, are deeply rooted, and are remarkably stable over time. Personality traits shape attitudes and beliefs, and have been found to significantly predict a wide range of behaviours across different contexts, including social interactions, information seeking, panic buying and hoarding, work performance, health risk behaviours and compliance with health guidance (Furr, 2009). Significantly, the influence of personality on behaviour is amplified in situations characterised by uncertainty or crisis, such as during a global pandemic (Caspi and Moffitt, 1993).

Models of basic personality structure provide a guide for research into individual differences in prosocial behaviours. One such model that has gained much attention in recent years is the HEXACO model of personality (Ashton et al., 2014). This model considers the domain of personality variation in terms of six basic traits: Honesty–Humility, Emotionality, eXtraversion, Agreeableness, Conscientiousness and Openness (hence, the acronym HEXACO). Honesty-Humility is a central factor of the HEXACO model and is described as ‘the tendency to be fair and genuine in dealing with others, in the sense of cooperation with others even when one might exploit them without suffering retaliation’ (Ashton and Lee, 2007). It is measured with the facets Sincerity, Fairness, Greed Avoidance and Modesty. Honesty-Humility appears to predict prosocial behaviours across a wide range of situations (Zettler and Hilbig, 2015). It has been suggested that the relationship between honesty-humility and prosocial behaviour is mediated separately and sequentially through perspective taking (the process of understanding thoughts and feelings from the other’s perspective) and guilt-proneness (a predisposition to experience negative feelings about personal wrongdoing, even when the wrongdoing is private; Fang et al., 2019).

Another important personality variable associated with prosocial behaviours is empathy, defined as ‘an affective state that stems from the apprehension of another’s emotional state or condition, and that is congruent with it’ (Eisenberg and Miller, 1987). Empathy is recognised as a multifaceted construct with three primary components: (a) an affective response to another person which often involves sharing that person’s actual or inferred emotional state; (b) a cognitive capacity to recognise and understand another’s emotional state; and (c) emotional regulatory mechanisms that keep track of the origins of self-and other-feelings (Decety and Jackson, 2004). Conceptualising empathy as an ability or capacity (i.e. empathy as a trait) implies that some individuals are more empathic than others (trait influence). However, empathy may also be context or situation-specific (state influence). Overall, the evidence suggests that empathy is a result of the interaction between state and trait influences (Cuff et al., 2016).

An individual-difference measure of empathy, the Interpersonal Reactivity Index (IRI) identifies two cognitive empathy processes (i.e. Perspective Taking and Fantasy) and two affective empathy processes (i.e. Empathic Concern and Personal Distress; Davis, 1983). Previous research strongly suggests that both affective as well as cognitive empathy processes motivate prosocial behaviours (Eisenberg et al., 2010). Specifically, Empathic Concern has been shown to promote altruism and caring behaviours (Batson, 2010; Sassenrath et al., 2016) while cognitive empathy processes have been associated with reductions of prejudice and intergroup conflict (Eisenberg et al., 2010; Klimecki, 2019; Todd and Burgmer, 2013). Findings from studies that have examined the relationships between different components of empathy and the HEXACO model of personality suggest that Empathic Concern is uniquely and positively linked with Honesty-Humility, Emotionality and Agreeableness, and cognitive empathy is associated with Agreeableness and Openness (Brazil et al., 2023; Romero et al., 2015).

Research conducted during the COVID-19 pandemic have reported that personality traits known to account for individual variation in prosocial behaviours predict compliance with COVID-19 TRBs (Aschwanden et al., 2021; Bacon et al., 2022; Miguel et al., 2021). A review and meta-analysis study found that people with low levels of Honesty-humility were less likely to engage in COVID-19 TRBs, and non-health-related prosocial behaviours (Ścigała et al., 2021). Other studies show that higher levels of Honesty-Humility predicted increased compliance with hand-washing and physical distancing guidance (Blais et al., 2021; Costantini et al., 2022; Kaufmann et al., 2022). Affective empathy for people vulnerable to COVID-19 has been shown to predict compliance with various COVID-19 TRBs, for example, hand washing (Kaufmann et al., 2022), physical distancing (Christner et al., 2020; Galang et al., 2021; Kaufmann et al., 2022; Pfattheicher et al., 2020), self-isolation (Petrocchi et al., 2021), wearing of face masks (Pfattheicher et al., 2020) and getting vaccinated against COVID-19 (Pfattheicher et al., 2022). It is relevant to note here that in the aforementioned studies, affective empathy was measured using the three-item Empathic Concern sub-scale of the IRI (Davis, 1983), and the items were specifically embedded in the context of COVID-19. Other studies (using multi-dimensional measures of empathy) found that trait empathy correlates with compliance with COVID-19 physical distancing regulations (Galang et al., 2021) and various COVID-19 TRBs (Morstead et al., 2022).

Success of a mass testing programme requires individual participation and commitment. Hence understanding the factors that can explain individual differences through applied behavioural science is potentially important to help with efforts to increase compliance. For these purposes, it is necessary to identify individual-level factors that may account for non-compliance with guidance on testing and reporting of LFT results. To our knowledge, there are no studies to date that have explored whether prosocial personality traits influence falsification of home COVID-19 LFTs.

Informed by the existing body of research, we hypothesised that an individual’s attitudes towards and intentions to engage in falsification of COVID-19 LFTs will be related to their levels of Honesty-Humility and affective empathy for people vulnerable to COVID-19. We conducted a two-study survey research to examine falsification of home LFTs in England during the COVID-19 pandemic. In study one, we estimated the prevalence of LFT falsification behaviours (FBs; using direct and indirect questioning methods) and explored psychosocial predictors of the behaviours (Ray et al., 2024). In study two (this study), we examined whether people’s attitudes towards, and intentions to engage with LFT FBs are influenced by (a) affective empathy for people vulnerable to COVID-19 operationalised as Empathic Concern (i.e. ‘sympathy and compassion towards others in need or in distress’ (Davis, 1983); and (b) Honesty-Humility from the HEXACO model (Ashton et al., 2014).

## Methods

The study was approved by Newcastle University Research, Policy, Intelligence and Ethics Team (reference: 24446/2022 dated 8 July 2022). We have complied with all relevant ethical regulations. Online informed consent was obtained from all study participants. Recruitment of participants and delivery of the online surveys for both studies were managed by YouGov^®^, a market research company. We selected YouGov (2021) as they have access to a panel of registered members that is representative of British adults in terms of age, sex, social class and education. Quota sampling was used to generate a nationally representative sample that met the eligibility criteria of this study. YouGov survey respondents receive points for completion of surveys (a short survey such as this one would be awarded 100 points), and once they achieve 5000 points, they receive a monetary award of £50.

### Participants

Eligible participants were adults (18 years and older) living in England who had previously taken a home LFT for COVID-19 and had completed the survey for study one (between 23rd and 30th September 2022). All YouGov registered panel members are assigned a unique respondent ID. This ID was used to identify respondents who completed the first stage of the survey and allowed us to invite only those respondents to the second study, without the need for processing any other identifiable information. All the statistical tests presented in this study were conducted on a sample size of 1295 participants. The details of the characteristics of survey respondents are presented in Supplemental Material, Table S1. Data for this study were collected between 14th October and 7th November 2022.

### Design

Responses to questions about LFT FBs are likely to be affected by social desirability bias in view of the moral and social implications of nonadherence to guidance (Tourangeau and Yan, 2007). Factorial survey designs are useful to assess sensitive topics as they are less prone to social desirability bias than conventional surveys (Walzenbach, 2019), and provide high internal validity (Eifler and Petzold, 2020). In a factorial survey, participants respond to short descriptions of hypothetical or real-life situations that are used as a stimulus to examine the effects of the characteristics of the situation on normative judgements, attitudes, behavioural intentions (Mutz, 2011) and/or simulated behaviours (Hrisos et al., 2008).

### Survey development

We developed four vignettes to represent the four COVID-19 LFT FBs that were examined in study one. All the vignettes are presented as Supplemental Material (Figures S1–S4).

*Vignette 1:* Reporting a positive test result as negative (FB *A*).
*Vignette 2:* Reporting a positive test after having produced a fake positive test (e.g. by
adding liquids such as a soft drink or other drink; FB *B*).
*Vignette 3:* Sharing test information of the LFT in order for someone else to report as their own (e.g. giving them the test strip ID or a picture of the test strip; FB *C*).
*Vignette 4:* Reporting a negative test without doing a test (FB *D*).

**Figure 1.**
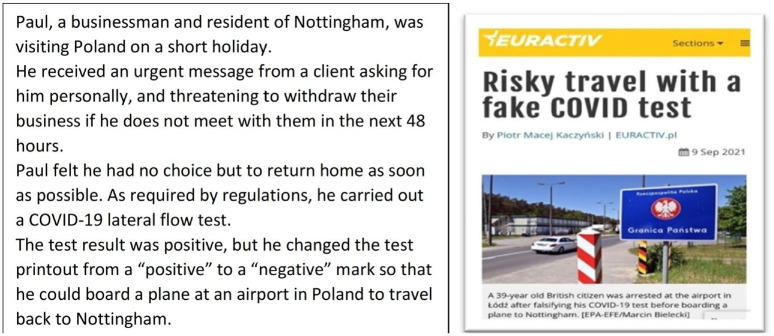
Vignette representing the falsification behaviour *A:* reporting a positive test result as negative.

Each vignette contained an image and a brief text description of a scenario based on published online media reports. The vignettes were developed with contributions from two members of the patient and public involvement strategic group of this research unit and subsequently refined following successive rounds of piloting by members of the public (volunteers) and work colleagues. As an example, Figure 1 shows vignette one.

Respondents were asked to assume that the guidance for reporting results of COVID-19 LFTs (DHSC, 2021) had not changed since April 2021. We chose a within-subject design (Auspurg et al., 2014) as the objective was to present each respondent with the same set of four vignettes. We randomised the order of the vignettes across the respondents, to minimise the impact of order effects on evaluation of the scenarios (Auspurg and Jäckle, 2017).

### Measures

The outcome measures were attitude and intention. Derived from the Theory of Planned Behaviour (Ajzen, 1991), we operationalised attitude as the degree to which a person considers it is acceptable (or not) for them to engage in COVID-19 LFT FBs. Intention was operationalised as the likelihood of a person to (not) engage in falsification of LFTs. Respondents rated the acceptability of the behaviour (attitude) depicted in each vignette, and their willingness to engage in that behaviour (intention), using 5-point Likert scales (for acceptability: 1 = totally acceptable, 5 = totally unacceptable; and for intention, 1 = very likely, 5 = very unlikely).

We measured affective empathy for people vulnerable to COVID-19 (for the purpose of brevity, hereafter referred to as Empathy in this text) by means of three items that were developed based on the Empathic Concern scale of the IRI (Davis, 1983). The Empathic Concern scale assesses ‘other-oriented’ feelings of sympathy and compassion towards others in need or in distress. This scale was recently tested in six related studies (Pfattheicher et al., 2019), and subsequently applied in COVID-19 research (Christner et al., 2020; Kaufmann et al., 2022; Petrocchi et al., 2021; Pfattheicher et al., 2020, 2022). The items applied in this study were: ‘I am very concerned about those most vulnerable to coronavirus (COVID-19)’; ‘I feel compassion for those most vulnerable to coronavirus (COVID-19)’, and ‘I am quite moved by what could happen to those most vulnerable to coronavirus if they contact COVID-19 infection’ (Supplemental Material, Figure S5). The scale showed high internal consistency (Cronbach’s alpha α = 0.91; it was 0.81 in the original study (Pfattheicher et al., 2019)). The scale’s response options ranged from 1 (‘strongly disagree’) to 5 (‘strongly agree’) with higher scores indicating greater Empathy.

We measured Honesty-Humility using four facet-level items included in this domain from the six-domain, 24-item Brief HEXACO Inventory (Ashton et al., 2014) answered on a 5-point Likert scale (1 = strongly disagree, 5 = strongly agree); an example item was ‘I find it difficult to lie’. The full scale is available in Supplemental Material (Figure S6). The Honesty-Humility scale showed low internal consistency (α = 0.42) accompanied by wide confidence intervals [0.96 to −1.9] within our sample. Hence, we used the four facet-level items of the Honesty-Humility domain (sincerity, modesty, fairness and greed avoidance) as single item measures in the analysis. Modesty was reverse coded as the statement used for this item was worded in a way that meant *lower* scores indicated *higher* levels of modesty, as opposed to the wording of the statements used for the other three items wherein higher scores indicated higher levels of those characteristics.

### Statistical analysis

Data analysis was conducted using R (version 4.2.0; https://www.R-project.org/). Scores for attitudes and intentions across the four vignettes were pooled to obtain a final attitude score and an intention score towards falsification behaviours. We conducted Spearman’s rank correlation between the study variables to account for the rank-based nature of the variables and check for multicollinearity. Next, we conducted stepwise (forward) regression between the outcome variables (attitudes and intentions) and the predictors (Empathy and the fours facets of Honest-Humility) with the aim to identify the most influential predictors that affect both the outcome variables. The model approach taken was one where the control variables, including age, sex and education, were entered first, followed by the candidate predictor variables – Empathy and the Honesty-Humility items. We conducted a multivariate stepwise regression with the ‘StepReg’ package (Li et al., 2024). This approach allows predictors to be added or removed algorithmically based on chosen criteria, ensuring that only the most influential variables that affect *both* the dependant variables are selected and ordered according to their impact on the dependant variable (Li et al., 2024). This procedure was adopted as it is computationally efficient and reduces the risk of overfitting. Furthermore, we believe this is the best approach for our research objective – which is identifying variables that are common between both the dependant variables, for better understanding of the behaviours and translation into potential policy recommendations.

We chose the Akaike information criterion (AIC) as our primary model selection tool because it provides a balance between model fit and complexity (Cavanaugh and Neath, 2019). While *R*^2^ measures the proportion of variance explained by the model, it tends to increase with the addition of more predictors, leading to model overfitting. AIC, on the other hand, penalises the inclusion of additional variables in the model, which makes it more appropriate for model selection (Hurvich and Tsai, 1989). The information regarding the selection process under AIC is provided in the Supplemental Materials (Table S2). In conjunction, we conducted univariate stepwise regression using the ‘StepReg’ package that permits use of adjusted *R*^2^ as the metric for model selection – this was done to obtain a holistic view of model performance. These results are displayed in the Supplemental Materials (Tables S3, S4A, and S4B). For the purpose of our study, we have opted for the multivariate stepwise regression to identify the common predictors using AIC as the model selection tool, as explained above.

Following the identification of predictors for best predictive performance, we estimated the effect sizes, as measured by Cohen’s *f*. Empathy was mean centred prior to the analysis. Attitude and intention scores were first regressed on Empathy, and then on the four facets of Honesty-Humility, respectively, while controlling for the demographic variables of age, sex and education. All statistical tests were evaluated at the significance level of α < 0.05.

## Results

The correlation matrix showed that none of the predictor variables were highly correlated with one another, which means that our regression models were not affected by the issue of multicollinearity as shown in Table 1 below. The model selection method identified age, Empathy (i.e. affective empathy for people vulnerable to COVID-19 infection), sincerity, fairness and modesty as the independent variables that provide the best predictive performance for both the response variables – attitude and intention. Specifically, higher scores for Empathy and the three facet-level items of Honest-Humility (sincerity, fairness modesty) predicted non-acceptability of the falsification behaviours and low intention to engage in them. It is to be noted that modesty was not statistically significant for intention (Table 2). Additionally, it was observed that greed avoidance was regressed out of the model as the stepwise regression did not identify it as an influential predictor for either of the outcome variables, as shown in Table 2.

**Table 1.** Spearman’s rank correlation matrix between all study variables, outcome and predictor variables of interest (*N* = 1295).

Variable	Attitude	Intention	Empathy	Sincerity	Modesty	Fairness	Greed
Attitude	1.00***	1.00***	0.38***	0.17***	0.12***	0.19***	0.10***
Intention	0.78***	0.78***	0.39***	0.20***	0.10***	0.21***	0.13***
Empathy	0.38***	0.38***	1.00***	0.22***	0.15***	0.22***	0.19***
Sincerity	0.17***	0.17***	0.22***	1.00***	0.12***	0.28***	0.15***
Modesty	0.12***	0.12***	0.15***	0.12***	1.00***	0.19***	0.07**
Fairness	0.19***	0.19***	0.22***	0.28***	0.19***	1.00***	0.21***
Greed-avoidance	0.10***	0.13***	0.19***	0.15***	0.07**	0.21***	1.00***

*** *p* < 0.001. **p < 0.01. *p < 0.05.

**Table 2.** Multivariate stepwise (forward) regression analysis with attitude and intention (*N* = 1295).

Outcome measure	Predictor	Beta coefficient	Std. Error	*t*-Value	*p*-Value	Adj. *R*^2^
Attitude	Empathy	0.449	0.032	13.97	<0.001***	0.223
	Age	0.104	0.015	6.546	<0.001***	
	Sincerity	0.066	0.024	2.695	0.007**	
	Fairness	0.044	0.021	2.079	0.037*	
	Modesty	0.005	0.027	2.113	0.034*	
Intention	Empathy	0.421	0.031	13.20	<0.001***	0.236
	Age	0.112	0.015	7.126	<0.001***	
	Sincerity	0.119	0.024	4.883	<0.001***	
	Fairness	0.055	0.021	2.609	0.009**	
	Modesty	0.039	0.027	1.436	0.151	

*** *p* < 0.001. **p < 0.01. *p < 0.05.

We proceeded to estimate effect sizes for the identified predictors, as measured by Cohen’s *f* (small = 0.14, medium = 0.39, large = 0.59). The effect size estimation in the intention model for Empathy was *f* = 0.48, indicating a medium effect. Age and sincerity observed a small effect with *f* = 0.22 and *f* = 0.16, respectively. Fairness and modesty both displayed weak effects (*f* = 0.08 and *f* = 0.04). Similarly, the effect sizes in the attitude model for Empathy was medium (*f* = 0.49), small for age (*f* = 0.20) and weak for sincerity, fairness and modesty (*f* = 0.09, 0.06 and 0.06, respectively).

## Discussion

The present study makes a unique contribution to the growing body of literature on predictors of (non)adherence to guidance for COVID-19 TRBs by highlighting the role of prosocial personality traits as predictors of falsification of at-home COVID-19 LFTs. Using a large quota-representative sample of adults living in England, this study is the first to report that affective empathy for people vulnerable to COVID-19) and Honesty-Humility (specifically, sincerity, modesty and fairness) are associated with positive attitudes (non-acceptability of FBs) and intentions to not engage with COVID-19 LFT FBs. However, according to effect sizes, as estimated by Cohen’s *f* in this study, empathy for people vulnerable to COVID-19 showed significant correlation for both attitude and intention, whereas the Honesty-Humility facet level measures displayed considerably weaker effects. Our findings are similar to those reported by a recent study (Kaufmann et al., 2022) that examined the links between these two personality traits and adherence to hand washing and physical distancing during the pandemic.

The findings of this study must be considered in the context of the findings of study one (same participant pool and for which data collection was completed 2 weeks prior to the start of data collection for this study) which showed that a proportion of the adult population in England engaged in falsification of at-home LFTs during the pandemic. For instance, when asked indirectly, an estimated 18.4% of people engaged in the FB ‘reporting a negative test without doing a test’ (depicted in vignette 4 in this study); however, when asked directly, only 5.73% of respondents reported having engaged in this FB (Ray et al., 2024).

The study findings are consistent with those of existing research on the role of affective empathy for people vulnerable to COVID-19 (Petrocchi et al., 2021; Pfattheicher et al., 2020) and Honesty-Humility (Modersitzki et al., 2021; Zettler et al., 2022) as promoters of compliance with various TRBs during the COVID-19 pandemic. Other research conducted during the COVID-19 pandemic suggests that levels of affective and cognitive empathy (specifically, empathic concern and perspective taking) and, correspondingly, prosocial tendencies might fluctuate in accordance with anxiety linked to risk exposure and risk perception (Cao et al., 2022; van de Groep et al., 2020). If COVID-19 LFTs FBs are considered risk taking behaviours, this perspective can provide insight into how these behaviours may be related to interactions between perception of risk of COVID-19, Empathy and Honesty-Humility. There is evidence that people with lower scores for Honesty-Humility perceive COVID-19 as a less serious risk than those with higher scores, and are less likely to adhere to various COVID-19 TRBs (Zettler et al., 2022). Further, there is evidence that when perception of risk is low, higher levels of empathic responding is associated with increased engagement with COVID-19 TRBs (Morstead et al., 2022).

A strength of this study is that it used a large nationally representative sample of the adult population who met the eligibility criteria. The sample was nationally balanced for age and sex: two key demographic characteristics that have been found to be associated with levels of empathy in UK adults (Sommerlad et al., 2021). A limitation of the study is that the results are based on self-reports in a cross-sectional survey. Falsification of test results during an ongoing pandemic is a sensitive issue. Responses to questions about acceptability of LFT FBs and intentions, as well as those measuring empathy and honesty-humility may have been influenced by social desirability bias (Müller and Moshagen, 2019) though the survey design included features that are believed to improve the validity of responses and reduce bias. Another limitation of this study is that the data for the present study was collected at a time when the UK government had lifted all forms of official restrictions, including the guidance for testing, and reporting results of home LFTs. Studies conducted in the UK (Schneider et al., 2021; Wright et al., 2022) and elsewhere (Franzen and Wöhner, 2021) have reported that prosocial attitudes, risk perceptions and uptake of COVID-19 TRBs varied between different time points during the pandemic. Although all respondents were provided with the instruction ‘please assume that the guidance for reporting results of a COVID-19 rapid LFT has not changed since April 2021’, it is possible that participants may have responded differently if this study was conducted during the period when LFT testing was being used to sanction activities, including social entertainment and travel. Furthermore, the study employed regression on Likert scale data, which is generally considered ordinal in nature. However, most parametric tests, such as the regression are robust to violations of these assumptions as shown in prior literature (Norman, 2010).

## Conclusion

### Implications for public health communications

It is important to consider that the evidence presented in this study is correlational and not experimental data. The findings are therefore associations, and we cannot claim causality. Nevertheless, the findings have implications for policy and public health communications not only during an ongoing pandemic but also in wider public health practice. The findings imply that prosocial appeals have the potential to be associated with greater compliance if they induce concern for the vulnerable (i.e. affective empathy). Thus, the study findings highlight the importance of focusing on affective empathy and prosocial attitudes at the centre of public health communications in health promotion and protection initiatives. Evidence synthesised from existing research suggests that prosocial public health communications that appeal to people’s other-oriented emotions (feeling concern for the other), especially those focusing on protecting friends and family, and tailoring those communications by taking into account individuals’ perceptions of risks and susceptibility are likely to positively impact on personal protective behaviours (Grimani et al., 2021). Incorporating empathy, sincerity and fairness into public health communications is important not only for normative ethical reasons, but also to build and maintain trust between members of the public, healthcare providers and authorities (Nihlén Fahlquist, 2018).

## Supplemental Material

sj-docx-1-hpq-10.1177_13591053241298034 – Supplemental material for Exploring personality correlates of falsification of COVID-19 lateral flow tests through vignettes Supplemental material, sj-docx-1-hpq-10.1177_13591053241298034 for Exploring personality correlates of falsification of COVID-19 lateral flow tests through vignettes by Devashish Ray, Raenhha Dhami, Aritra Mukherjee, Jan Lecouturier, Laura J McGowan, Ivo Vlaev, Michael P Kelly and Falko F Sniehotta in Journal of Health Psychology

sj-jpg-2-hpq-10.1177_13591053241298034 – Supplemental material for Exploring personality correlates of falsification of COVID-19 lateral flow tests through vignettes Supplemental material, sj-jpg-2-hpq-10.1177_13591053241298034 for Exploring personality correlates of falsification of COVID-19 lateral flow tests through vignettes by Devashish Ray, Raenhha Dhami, Aritra Mukherjee, Jan Lecouturier, Laura J McGowan, Ivo Vlaev, Michael P Kelly and Falko F Sniehotta in Journal of Health Psychology

sj-jpg-3-hpq-10.1177_13591053241298034 – Supplemental material for Exploring personality correlates of falsification of COVID-19 lateral flow tests through vignettes Supplemental material, sj-jpg-3-hpq-10.1177_13591053241298034 for Exploring personality correlates of falsification of COVID-19 lateral flow tests through vignettes by Devashish Ray, Raenhha Dhami, Aritra Mukherjee, Jan Lecouturier, Laura J McGowan, Ivo Vlaev, Michael P Kelly and Falko F Sniehotta in Journal of Health Psychology

sj-jpg-4-hpq-10.1177_13591053241298034 – Supplemental material for Exploring personality correlates of falsification of COVID-19 lateral flow tests through vignettes Supplemental material, sj-jpg-4-hpq-10.1177_13591053241298034 for Exploring personality correlates of falsification of COVID-19 lateral flow tests through vignettes by Devashish Ray, Raenhha Dhami, Aritra Mukherjee, Jan Lecouturier, Laura J McGowan, Ivo Vlaev, Michael P Kelly and Falko F Sniehotta in Journal of Health Psychology
