# Supplementary material for: Exploring personality correlates of falsification of COVID-19 lateral flow tests through vignettes
Source: J Health Psychol. 2024 Nov 25;30(10):2529–41. doi: 10.1177/13591053241298034 (PMC12381394; doi:10.1177/13591053241298034)
Supplement: sj-docx-1-hpq-10.1177_13591053241298034 – Supplemental material for Exploring personality correlates of falsification of COVID-19 lateral flow tests through vignettes [file sj-docx-1-hpq-10.1177_13591053241298034.docx]

**Title**: Exploring personality correlates of falsification of COVID-19 lateral flow tests through vignettes.

**Supplementary Table S1**: Demographic characteristics of survey participants (N = 1,295)

| **Category** | Frequency (percentage in parentheses) | | Average for England and Wales, 2021 (percentage) |
| --- | --- | --- | --- |
| **Sex** | *Male* | 556 (42.9) | 49 |
|  | *Female* | 739 (57) | 51 |
| **Gender identity** | *Cis* | 1250 |  |
|  | *Trans (including non-binary)* | 8 |  |
| **Age (in Years)** | *18-24* | 109 (8.4) | 11 |
|  | *25-34* | 264 (20.3) | 17 |
|  | *35-44* | 241 (12.35) | 16 |
|  | *45-54* | 226 (17.4) | 17 |
|  | *55-64* | 236 (18.2) | 15 |
|  | *65+* | 219 (16.9) | 23 |
| **Social Grade*** | *A, B, C1* | 855 (66) | 56.2 |
|  | *C2, D, E* | 440 (34) | 43.8 |
| **Ethnicity** | *White* | 1140 (90.5) | 81.7 |
|  | *Ethnic minority* | 144 (11.1) | 18.2 |
|  | *Not available* | 11 |  |
| **Education** | *High (University degree)* | 586 (45.2) | 33.8 |
|  | *Medium (College or University diploma)* | 481(37.1) |  |
|  | *Low (GCSE or lower)* | 228 (17.6) |  |
| **Region in England** | *Northeast* | 66 |  |
|  | *Northwest* | 161 |  |
|  | *Yorkshire and Humber* | 127 |  |
|  | *East Midlands* | 135 |  |
|  | *West Midlands* | 117 |  |
|  | *East of England* | 144 |  |
|  | *London* | 140 |  |
|  | *Southeast* | 256 |  |
|  | *Southwest* | 149 |  |

* *A=upper middle class, B=middle class, C1=lower middle class, C2= skilled working class, D=working class, E= those at lowest level of subsistence*. The social grade definitions by the National Readership Survey (https://nrs.co.uk/) are widely used as a generic reference series for classifying and describing social classes, especially for consumer targeting and consumer market research by the advertising UK media and publishing sectors.

**Supplementary Table S2** Table. Summary of selection process under forward with AIC for multivariate stepwise regression – model selection identifies most influential predictors affecting *both* the outcome variables.

| **Step** | **Variable selected** | **AIC** |
| --- | --- | --- |
| **1** | Intercept | 9397.09 |
| **2** | Empathy | 9106.97 |
| **3** | Age | 9045.12 |
| **4** | Sincerity | 9016.97 |
| **5** | Fairness | 9013.05 |
| **6** | Modesty | 9012.56 |

**Supplementary Table S3.** Summary of selection process under forward univariate stepwise regression with adjusted R^2^ for attitude

| **Step** | **Variable selected** | **Adj R^2^** |
| --- | --- | --- |
| **1** | Intercept | 0 |
| **2** | Empathy | 0.183 |
| **3** | Age | 0.213 |
| **4** | Sincerity | 0.219 |
| **5** | Modesty | 0.222 |
| **6** | Fairness | 0.224 |

**Supplementary Table S4 A and S4 B**

**Table S4. A.** Univariate Stepwise (forward) regression analysis with intention (N = 1,295).

| **Outcome measure** | **Predictor** | **Beta coefficient** | **Std. Error** | **t-value** | **p-value** | **Adj. R^2^** |
| --- | --- | --- | --- | --- | --- | --- |
|  | Intercept | -1.359 | 0.163 | -8.337 | P<0.001*** |  |
| Intention | Empathy | 0.421 | 0.031 | 12.925 | P<0.001*** | 0.236 |
|  | Age | 0.112 | 0.015 | 7.513 | P<0.001*** |  |
|  | Sincerity | 0.119 | 0.024 | 4.661 | P<0.001*** |  |
|  | Fairness | 0.055 | 0.021 | 2.466 | 0.0138* |  |
|  | Education (Medium) | 0.134 | 0.071 | 1.885 | 0.059 |  |
|  | Education (High) | 0.148 | 0.070 | 2.112 | 0.035* |  |
|  | Modesty | 0.037 | 0.027 | 1.362 | 0.173 |  |
|  | Sex (Women) | 0.059 | 0.050 | 1.180 | 0.238 |  |

*** for p < 0.001, ** for p < 0.01, * for p < 0.05. The reference for Education was ‘low education’ level and the reference for sex was ‘Male.’

**Table S4. B.** Summary of selection process under forward univariate stepwise regression with adjusted R^2^ for intention******

| **Step** | **Variable selected** | **Adj R^2^** |
| --- | --- | --- |
| **1** | Intercept | 0 |
| **2** | Empathy | 0.176 |
| **3** | Age | 0.214 |
| **4** | Sincerity | 0.232 |
| **5** | Fairness | 0.236 |
| **6** | Education | 0.237 |
| **7** | Modesty | 0.238 |
| **8** | Sex | 0.238 |

******Model selection under adjusted R2 for ‘intention’ with univariate stepwise regression leads to the selection of all predictors added to the model. The mode fit also increases with the addition of each predictor. However, model selection under adjusted R2 for ‘attitude ‘remains commensurate to the multivariate stepwise regression using model selection tool AIC. We report model selection results under both approaches to provide a holistic overview for model comparison.

The preferred approach for our study was the multivariate stepwise regression, as this approach aims to identify common predictors between the two outcome variables, which would facilitate understanding and translation into potential policy recommendations.


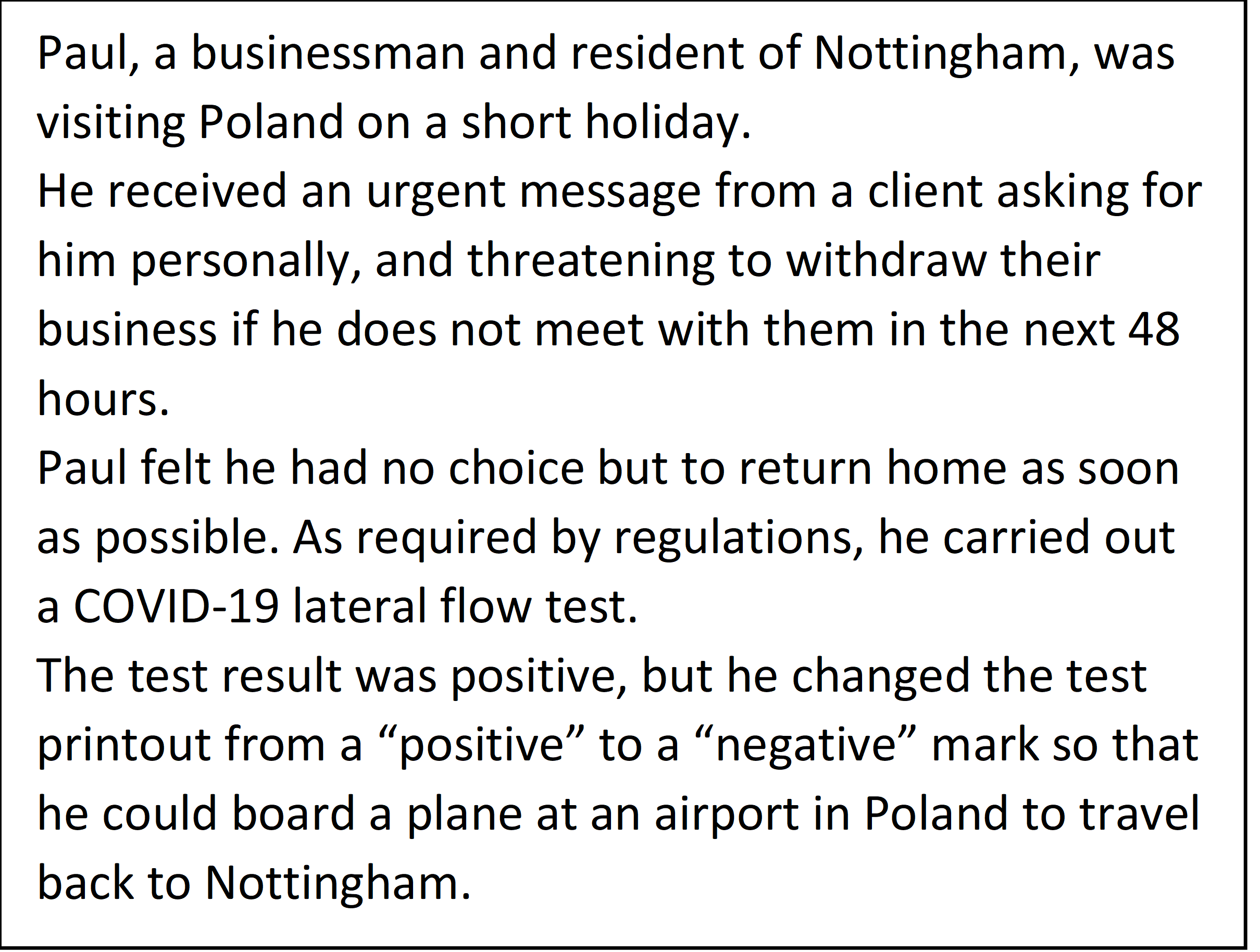

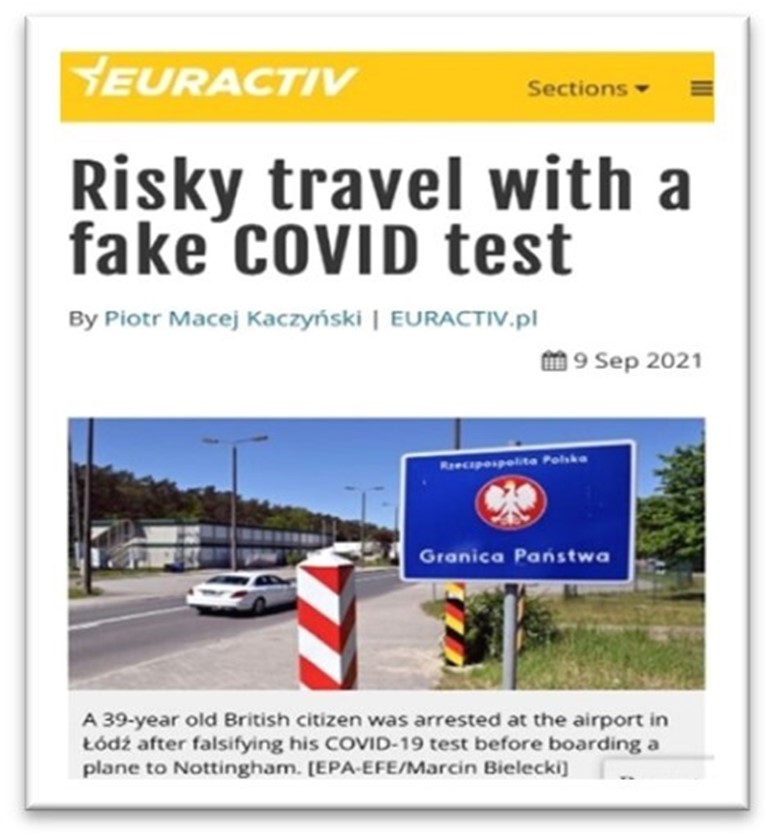


Figure S1. Vignette representing the falsification behaviour *A*: reporting a positive test result as negative.


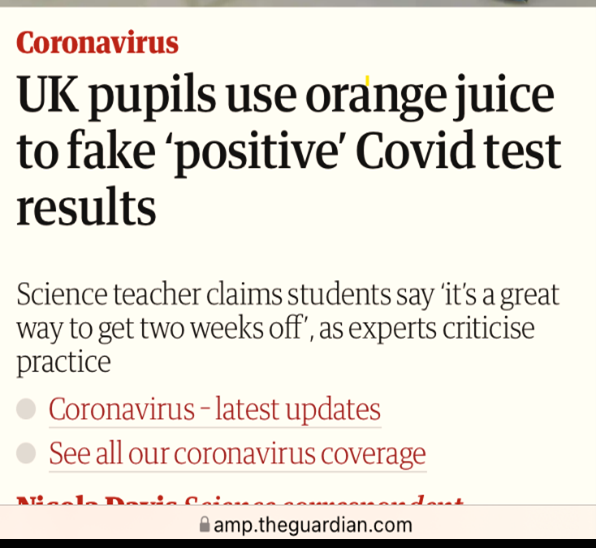


Lucy is a trainee doctor.

She has been feeling very stressed due to the combined pressures of working and studying which involves spending long hours in the hospital and revising in the evening.

She was desperate to have a couple of weeks off work. She had seen videos of young people using orange juice to fake a positive COVID-19 lateral flow test.

Lucy managed to produce a fake positive test result which allowed her to stay off work for a week.

Figure S2. Vignette representing the falsification behaviour *B*: Reporting a positive test after having produced a fake positive test

Sam was getting ready to go to a music concert where his favourite band were performing.

Just as he was about to leave, his best mate Bob rang to remind him that entry to the concert venue required proof of a recent negative lateral flow test.

Sam did not have any test kits at home.

To help out his friend, Bob shared the number of a test strip from the test pack he was using so that Sam could register it as a negative test result.


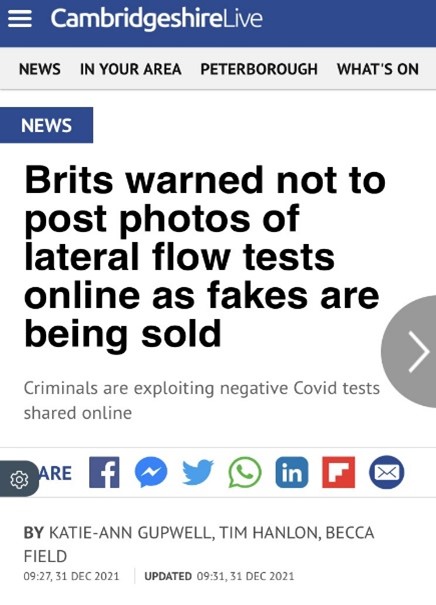


Figure S3. Vignette representing the falsification behaviour *C:*  Sharing information of the Lateral Flow test (e.g., test strip number)

In order for someone else to report as their own.

Jane works for a small company.

For the past several weeks she has been taking extra shifts to build up her savings.

Jane is required to register a negative test result before each shift. Recently, one of her colleagues who has no symptoms has tested positive and has been asked to self-isolate and report to work only after she tests negative.

Jane has no symptoms but is worried that if she too tests positive, she will need to self-isolate for at least a week and not be able to work.

Jane is concerned about loss of income and decides to register a negative test without actually doing the test and goes into work.


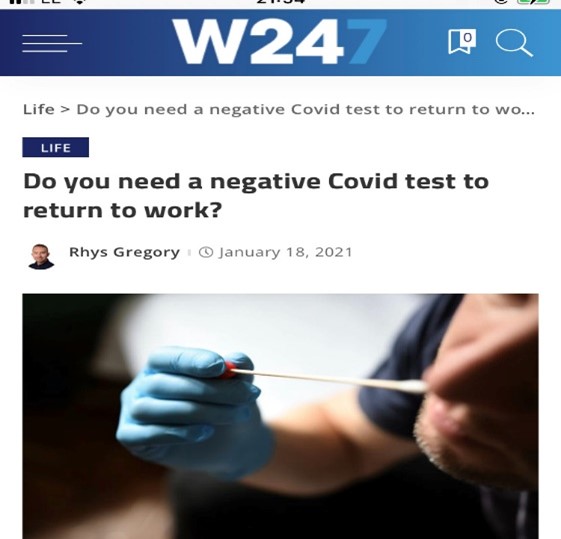


Figure S4. Vignette representing the falsification behaviour *D:* Reporting a negative test result without doing a test.

| **Q 1. I am very concerned about those most vulnerable to coronavirus (COVID-19)** |
| --- |
| Strongly disagree Disagree Neither agree nor disagree Agree Strongly agree |

| **Q 2. I feel compassion for those most vulnerable to coronavirus (COVID-19)** |
| --- |
| Strongly disagree Disagree Neither agree nor disagree Agree Strongly agree |

| **Q 3. I am quite moved by what could happen to those most vulnerable to coronavirus if they contract COVID-19** |
| --- |
| Strongly disagree Disagree Neither agree nor disagree Agree Strongly agree |

Figure S5. Three-item scale used to measure empathy for people vulnerable to COVID-19 (adapted from scale used in previous research as referenced in main manuscript).

| **Q 1. I find it difficult to lie** |
| --- |
| Strongly disagree Disagree Neither agree nor disagree Agree Strongly agree |
|  |
| **Q 2. I think I am entitled to more respect than the average person is** |
| Strongly disagree Disagree Neither agree nor disagree Agree Strongly agree |
|  |
| **Q 3. I wouldn’t pretend to like someone just to get that person to do favours for me** |
| Strongly disagree Disagree Neither agree nor disagree Agree Strongly agree |
|  |
| **Q 4. Having a lot of money is not especially important to me** |
| Strongly disagree Disagree Neither agree nor disagree Agree Strongly agree |

Figure S6. The **Honesty-Humility questionnaire** (adapted from scale used in previous research as stated in the main manuscript). Q1= Fairness; Q2= Modesty; Q3= Sincerity; Q4- Greed avoidance
