# Supplementary figures and images for: Exploring personality correlates of falsification of COVID-19 lateral flow tests through vignettes
Source: J Health Psychol. 2024 Nov 25;30(10):2529–41. doi: 10.1177/13591053241298034 (PMC12381394; doi:10.1177/13591053241298034)

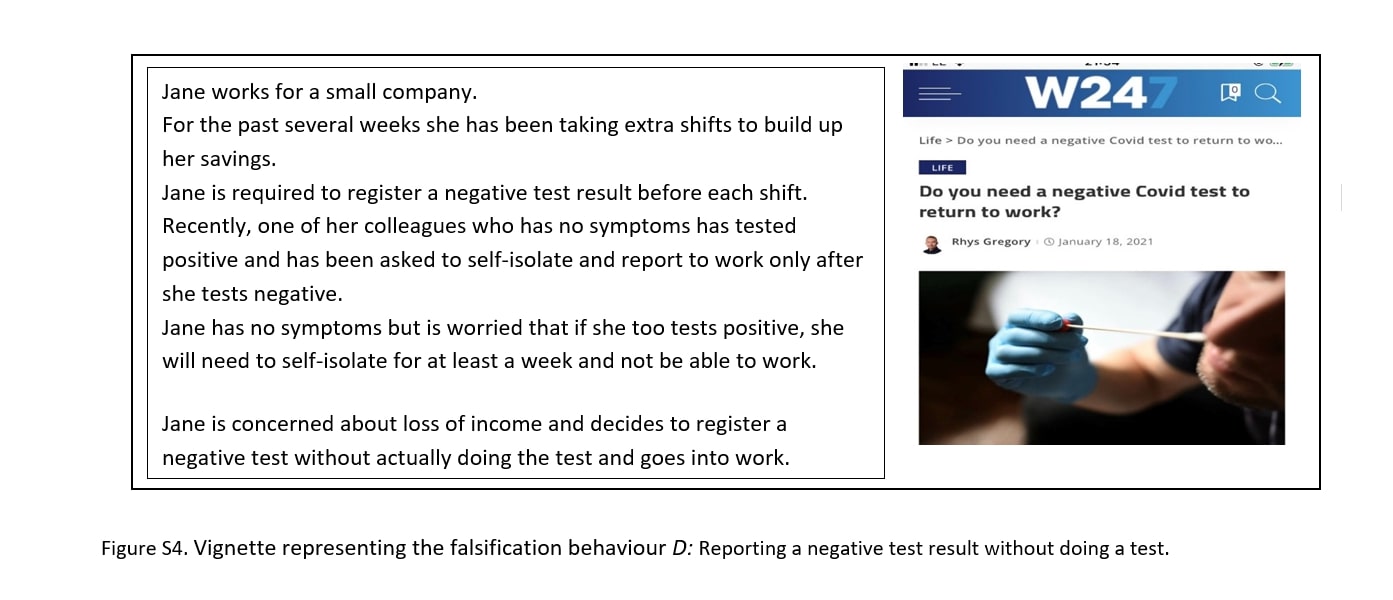

Supplement: sj-jpg-2-hpq-10.1177_13591053241298034 – Supplemental material for Exploring personality correlates of falsification of COVID-19 lateral flow tests through vignettes [file sj-jpg-2-hpq-10.1177_13591053241298034.jpg]

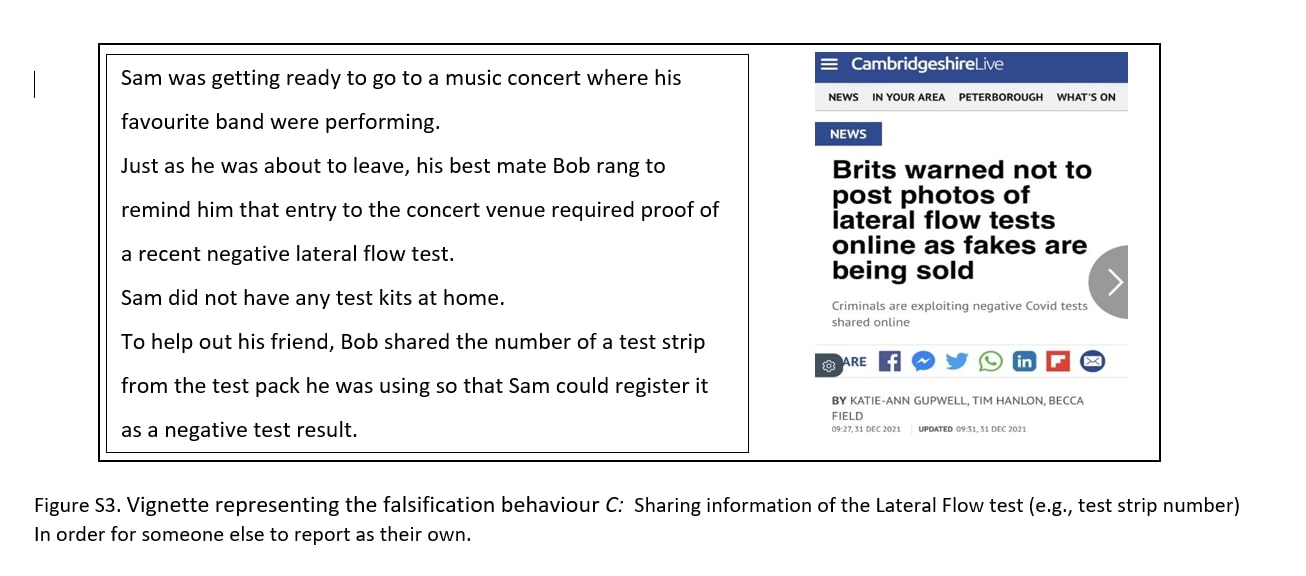

Supplement: sj-jpg-3-hpq-10.1177_13591053241298034 – Supplemental material for Exploring personality correlates of falsification of COVID-19 lateral flow tests through vignettes [file sj-jpg-3-hpq-10.1177_13591053241298034.jpg]

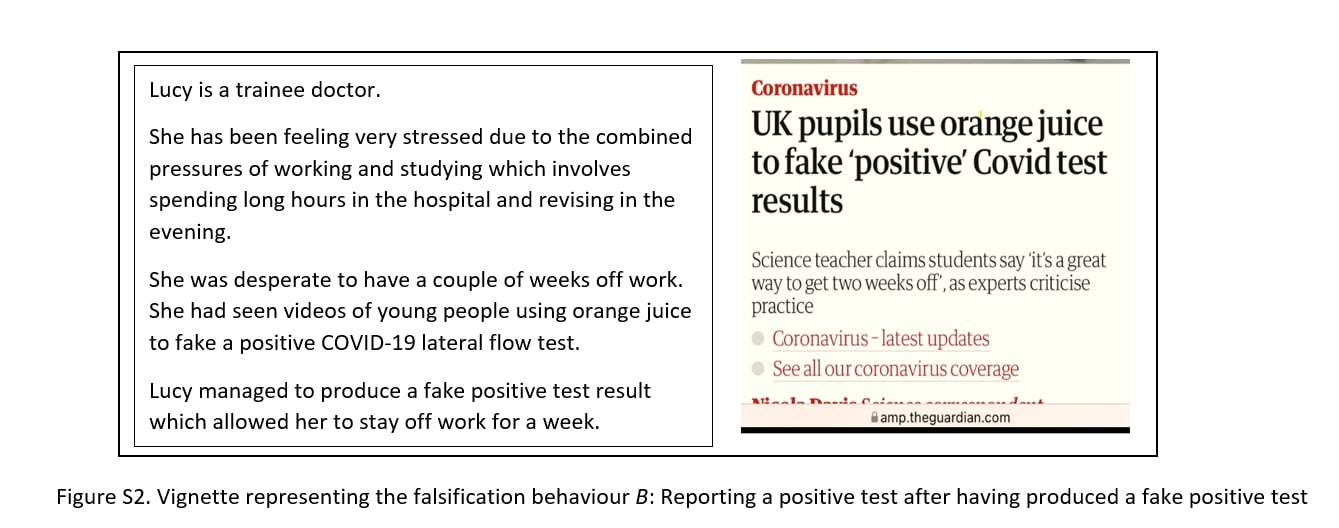

Supplement: sj-jpg-4-hpq-10.1177_13591053241298034 – Supplemental material for Exploring personality correlates of falsification of COVID-19 lateral flow tests through vignettes [file sj-jpg-4-hpq-10.1177_13591053241298034.jpg]
